# Antibiotics in ingested human blood affect the mosquito microbiota and capacity to transmit malaria

**DOI:** 10.1038/ncomms6921

**Published:** 2015-01-06

**Authors:** Mathilde Gendrin, Faye H. Rodgers, Rakiswendé S. Yerbanga, Jean Bosco Ouédraogo, María-Gloria Basáñez, Anna Cohuet, George K. Christophides

**Affiliations:** 1 Department of Life Sciences, Imperial College London, London SW7 2AZ, UK; 2 Institut de Recherche en Sciences de la Santé, 01 BP 545, Bobo-Dioulasso 01, Burkina Faso; 3 Department of Infectious Disease Epidemiology, School of Public Health, Imperial College London, London W2 1PG, UK; 4 Institut de Recherche pour le Développement, Unité MIVEGEC, 34394 Montpellier, France; 5 The Cyprus Institute, 2121 Nicosia, Cyprus

## Abstract

Malaria reduction is most efficiently achieved by vector control whereby human populations at high risk of contracting and transmitting the disease are protected from mosquito bites. Here, we identify the presence of antibiotics in the blood of malaria-infected people as a new risk of increasing disease transmission. We show that antibiotics in ingested blood enhance the susceptibility of *Anopheles gambiae* mosquitoes to malaria infection by disturbing their gut microbiota. This effect is confirmed in a semi-natural setting by feeding mosquitoes with blood of children naturally infected with *Plasmodium falciparum*. Antibiotic exposure additionally increases mosquito survival and fecundity, which are known to augment vectorial capacity. These findings suggest that malaria transmission may be exacerbated in areas of high antibiotic usage, and that regions targeted by mass drug administration programs against communicable diseases may necessitate increased vector control.

## Abstract (web summary)

The gut microbiota of malaria-transmitting mosquitoes contributes to the insects’ resistance to the parasite. Here, Gendrin *et al*. show that antibiotics in ingested human blood alter the mosquito gut microbiota and increase the insect’s survival, fecundity and susceptibility to the parasites.

## Graphical abstract

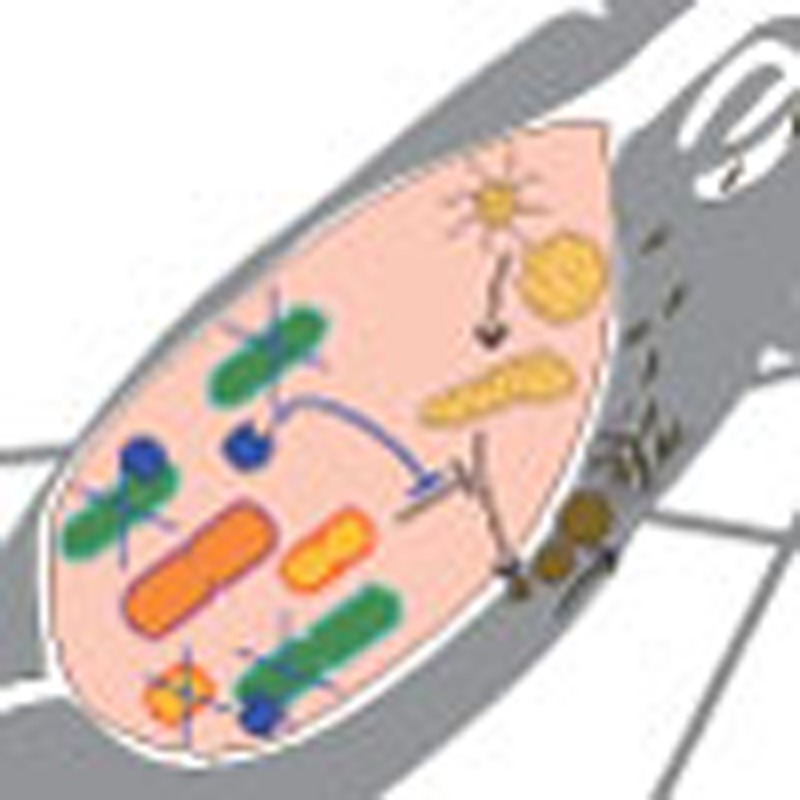

Malaria is caused by the parasite *Plasmodium*, transmitted via bites of *Anopheles* mosquitoes. *Plasmodium* gametocytes ingested with the blood produce gametes that undergo sexual reproduction inside the mosquito midgut^1^. Zygotes subsequently traverse the midgut epithelium to develop into oocysts on its basal side. After one to two weeks of multiplicative growth, the oocysts release thousands of sporozoites, which invade the salivary glands and can be transmitted to humans.

Concurrently with parasite uptake and development in the gut lumen, commensal gut bacteria proliferate in response to the blood meal^2^,^3^. This microbiota adversely affects parasite development both directly through the production of free radicals and indirectly through the induction of immune responses^4^,^5^,^6^,^7^. Parasite infection success increases if gut microbial colonisation is prevented throughout mosquito adulthood via feeding with an antibiotic-treated sugar solution^5^,^8^. Therefore, we hypothesised that antibiotics present in the blood meal of mosquitoes hosting a conventional microbiota could likewise interfere with bacterial proliferation, thus enhancing mosquito susceptibility to *Plasmodium* and promoting malaria transmission. Such an effect could considerably influence the malaria burden in sub-Saharan Africa where the use of antibiotics is high, particularly in areas implementing elimination programs of neglected tropical and other communicable diseases that require the use of antibiotics^9^,^10^,^11^,^12^,^13^,^14^,^15^.

Here, we tested our hypothesis by supplementing blood with therapeutic concentrations of antibiotics to the mosquito *A. gambiae* and analysing their effect on the microbiota and on several components of the mosquito vectorial capacity, namely susceptibility to *Plasmodium* infection, survival rate and fecundity. As our antibiotic model, we chose a cocktail of penicillin and streptomycin (PS) that is commonly used in mosquito and parasite research without any known effect on mosquitoes or parasites^8^,^16^,^17^,^18^,^19^, has broad antibacterial activity and does not require metabolic activation. We show that exposure of mosquitoes to this antibiotic cocktail significantly affects their gut microbiota, increases their susceptibility to *Plasmodium* infection and augments their survival and fecundity. These findings suggest that PS treatment of *Plasmodium* gametocyte carriers may increase the risk of malaria transmission.

## Results

### Effect of PS on the mosquito gut microbiota

**Figure 1:**
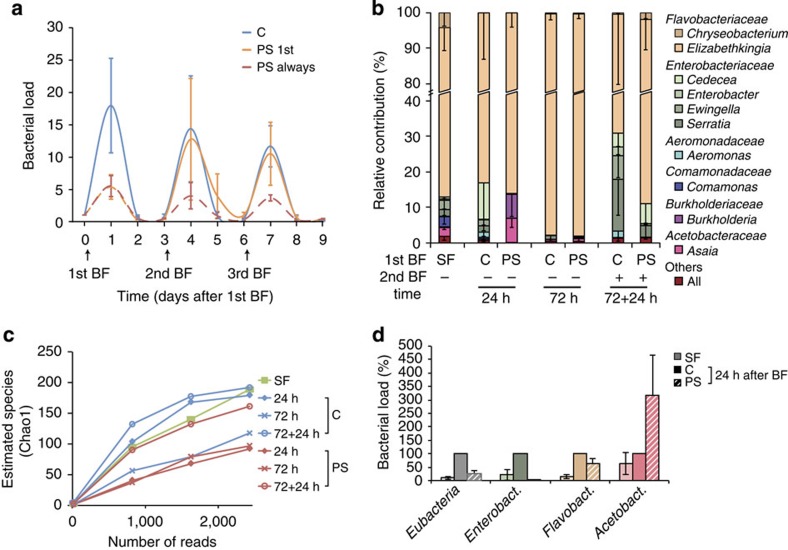
Blood meal antibiotics affect mosquito microbiota. (**a**) qPCR quantification of midgut bacterial 16S rDNA every 24 h during a three blood-feed (‘BF’) course. Blood was supplemented with penicillin/streptomycin (‘PS always’), water (control, ‘C’) or PS in the first blood meal and water thereafter (‘PS 1st’). (**b**) 16S rDNA 454-pyrosequencing analysis of mosquito gut microbiota before blood meal (sugar fed, ‘SF’), 24 and 72 h after PS-treated or untreated blood meal and 24 h after an untreated second blood meal. Main bacterial genera (>5% in at least one sample) and families are indicated. (**c**) Rarefaction curve of the estimated number of species using the Chao1 method. (**d**) qPCR microbiota analysis using generic or taxon-specific 16S rDNA primers. Data show average from three (**a**–**c**) to six (**d**) independent experiments, error bars show s.e.m., *n*=15–25 mosquitoes per sample and per replicate. qPCR data show fold versus day 0 (**a**) and % of control BF. (**d**) *Enterobact*.—*Enterobacteriaceae*; *Flavobact*.—*Flavobacteriaceae; Acetobact.*—*Acetobacteraceae.*

We monitored the bacterial load in the mosquito gut over a time course of three blood feeds offered every three days through an artificial skin. Quantitative PCR (qPCR) on the 16S rDNA revealed that the massive proliferation of bacteria seen at 24 h after the blood meal was reduced by 70% (*P*=0.034, analysis of variance (ANOVA) test following lmer, see methods) in the presence of PS (Fig. 1a), without any concurrent reduction in blood meal size (Supplementary Fig. 1a). This effect was recurrent upon PS exposure in all three blood meals. The addition of PS to only the first blood meal, recapitulating a more realistic feeding regimen on antibiotic and non-antibiotic users, did not persistently affect microbiota proliferation after the second and third blood meals (Fig. 1a).

We monitored the composition of the mosquito gut microbiota over a course of two blood feeds by 454-pyrosequencing of the 16S rDNA. The gut microbiota of our *A. gambiae* colony was dominated by *Flavobacteriaceae* and *Enterobacteriaceae* (Fig. 1b), consistent with previous findings in field-collected and laboratory-reared mosquitoes^4^,^20^,^21^,^22^. A high variability in microbiota composition was recorded between the replicates representing different mosquito batches (Supplementary Fig. 2): 45 of 78 genera were detected in only one of the three replicate experiments, while the relative abundance of genera varied greatly between the replicate samples, including 0–19.5% and 0–30.7% for *Burkholderia* and *Cedecea*, respectively.

PS supplementation into the blood affected the microbiota composition 24 h after a blood meal (Fig. 1b). Importantly, it halved the within sample microbiota diversity (alpha diversity) (Fig. 1c and Supplementary Table 1), reduced the proportion of *Enterobacteriaceae* 92-fold (*P*=0.23, ANOVA/lmer), and led to a 19-fold increase of *Acetobacteraceae* (*P*=0.017, ANOVA/lmer) solely represented by *Asaia sp.* (Fig. 1b). The effect on *Enterobacteriaceae* was confirmed by taxon-specific qPCR including more experiments (Fig. 1d; *P*=0.012, ANOVA/lmer). The proportion of *Flavobacteriaceae* was not affected by the PS treatment (Fig. 1b, *P*=0.79, ANOVA/lmer), which may be linked to the resistance of *Elizabethkingia* to PS^23^. After a second (non-PS treated) blood meal, the decrease in alpha diversity was still observed (Fig. 1c and Supplementary Table 1), but the effects on *Enterobacteriaceae* and *Acetobacteraceae* were much reduced (Fig. 1b; threefold decrease, *P*=0.33, and 8.7% decrease, *P*=0.55%, respectively, ANOVA/lmer).

### Impact of PS exposure on *Plasmodium* infectivity

**Figure 2:**
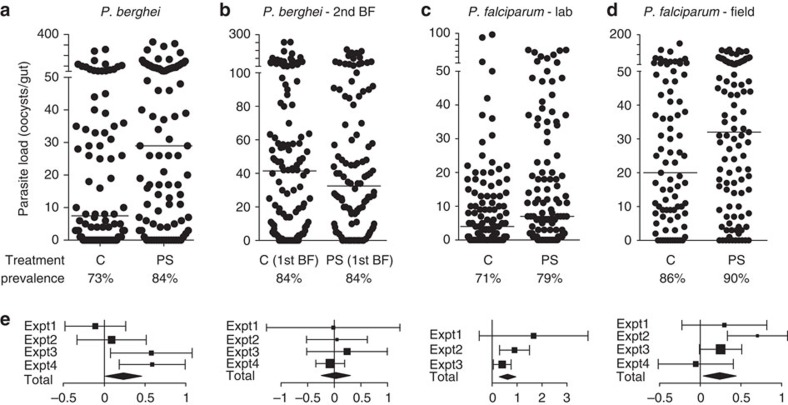
Antibiotics increase mosquito susceptibility to infection by *Plasmodium* ingested in the same blood meal. (**a**–**d**) Oocyst counts after PS-treated or control (‘C’) blood meal on *P. berghei*-infected mice (**a**), naive mice, followed with a 2nd blood meal on untreated P. berghei-infected mice 3 days later (**b**), *P. falciparum* gametocyte cultures (**c**) and naturally infected P. falciparum blood samples (**d**). Medians and oocyst distributions of representative experiments are shown. (**e**) Forest plots showing the effect of PS treatment on *Plasmodium* infection intensities in each replicate as determined by GLMM analysis. The variation of the fixed effect estimate in each (squares) and all (diamonds) replicates are shown (±95% confidence interval, glmmADMB). Within each chart, the square size is proportional to the mosquito number. From left to right, charts show the effect on *P. berghei* - 2nd BF, *P. falciparum*—lab and *P. falciparum*—field respectively. Mosquitoes (*n*=18–205) per experiment and per condition (average 86 (±8 s.e.m.)).

We examined whether the ingestion of PS-containing blood influences mosquito susceptibility to *Plasmodium*. Mosquitoes were fed on *Plasmodium berghei*-infected rodents before or following intravenous injection of PS, and the infection was monitored by counting the proportion of mosquitoes carrying oocysts (prevalence) and the number of oocysts per mosquito (intensity). Prevalence increased by 21% (*P*=5.10^−7^, ANOVA/glmer) and median intensity doubled (*P*=0.041, +24% of the fixed effect estimate; +108% of the median, Wald/glmmADMB) upon PS treatment (Fig. 2a,e). No effects on prevalence or intensity were observed when the infection was introduced in the second blood meal following an initial PS-treated non-infectious blood meal (Fig. 2b, e; *P*=0.5, Wald/glmmADMB). These results are consistent with the restoration of control microbiota load and, broadly, population composition following a second, non-PS treated, blood meal (Fig. 1a,b), supporting our hypothesis that the increase in *Plasmodium* infection is microbiota mediated.

To investigate the effects of PS on infection by human parasites, mosquitoes were fed on blood containing *in vitro* cultured *P. falciparum* gametocytes and supplemented with PS or buffer before blood feeding. Consistently with *P. berghei* infections, higher infection prevalence (*P*=0.0033, +138%, ANOVA/glmer) and intensity (*P*=0.00026, +62% of fixed effect estimate, +25% of the median, Wald/glmmADMB) were recorded when the infectious blood meal was supplemented with PS (Fig. 2c,e).

We examined the implications of these findings on human malaria transmission in Burkina Faso, in conditions close to field malaria transmission. *A. gambiae* from a recently colonised population^24^ were fed on blood freshly drawn from children carrying *P. falciparum* gametocytes and supplemented with PS or buffer. Four replicate experiments were performed, each using the blood from a different gametocyte carrier. The obtained data confirmed our rodent model and cultured parasite data and validated our hypothesis that the presence of antibiotics in the blood meal indeed impacts mosquito susceptibility to infection (Fig. 2d,e). The intensity of *P. falciparum* infection significantly increased in PS-exposed mosquitoes (*P*=0.02, +24% of the fixed effect estimate; +57% of the median, Wald/glmmADMB). A 6.3% increase in infection prevalence was not statistically significant (*P*=0.4, ANOVA/glmer), which is likely due to reduced statistical power of the experiment. It has been shown that whilst prevalence and intensity are clearly linked, effects on prevalence are more pronounced at low infection intensities, whereas effects on intensity are irrespective of the exposure level^25^. The high infection level observed in some of the replicates, averaging up to 29 oocysts in control mosquitoes (Supplementary Table 2), might have therefore masked the effect on prevalence.

### Impact of PS exposure on mosquito fitness

**Figure 3:**
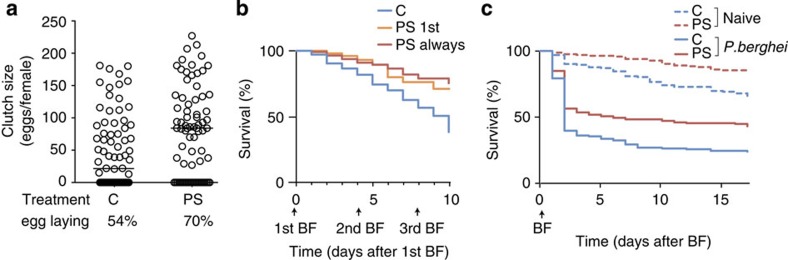
Blood meal antibiotics increase mosquito survival and fecundity. (**a**) Mosquito clutch size and percentage of females that laid eggs (‘Egg laying’) following PS-treated or control blood meal. Medians and distributions of egg numbers per female of one representative experiment of three are shown. (**b** and **c**) Kaplan–Maier survival plots of mosquitoes fed on human blood (**b**) or mice (**c**). In **b**, mosquitoes were offered three control blood meals (‘C’), three PS-treated blood meals (‘PS always’), or a PS-treated first blood meal and two control blood meals (‘PS 1st’). In **c**, mosquitoes were fed once on *P. berghei*-infected (solid lines) or naive (dashed lines) mice. Data are pooled from three (**b**) to four (**c**) independent experiments. Mosquitoes (*n*=50) per experiment and per condition.

Mosquito vectorial capacity is not only determined by susceptibility to the malaria parasites; mosquito lifespan, population size and human-biting rate also have strong bearings on malaria prevalence^26^. We therefore investigated whether the addition of PS to a blood meal influences mosquito reproduction and survival. Treatment led to increased fecundity (Fig. 3a), including a 32% higher proportion of egg-laying females (*P*=0.0038, ANOVA/glmer) and a 53% higher average number of eggs per female (*P*=0.016, ANOVA/lmer), while the proportion of eggs hatching into larvae was not affected (Supplementary Fig. 1b; *P*=0.79, ANOVA/glmer). This increased fecundity is likely to augment the size of mosquito populations, particularly when larval competition in breeding sites is low, notably at the beginning of the rainy season^27^. The mosquito survival rate also significantly increased following ingestion of PS-treated human blood (Fig. 3b; *P*=0.017 for PS in the first blood meal; *P*=0.0016 for PS in all three blood meals, log-rank test), or feeding on PS-treated mice, whether naive or *P. berghei*-infected (*P*=0.0098 and *P*=0.0029, respectively, Gehan–Wilcoxon test; Fig. 3c). Increased mosquito survival rate strongly affects vectorial capacity, by increasing the likelihood of infected mosquitoes surviving beyond the parasite extrinsic incubation period (time required between being ingested and becoming infective to humans), and therefore the probability of transmitting the disease to new hosts.

## Discussion

Our data show that the presence of PS, a wide-spectrum antibiotic cocktail, in *Plasmodium* gametocyte-containing blood enhances critical parameters affecting the ability of mosquitoes to transmit malaria, including the mosquito fitness and susceptibility to *Plasmodium* infection. Further epidemiological and modelling studies can quantify the outcome of these effects on malaria transmission. To date, research in aseptic mosquitoes has revealed various beneficial effects of microbiota on mosquito physiology; it accelerates development^28^, protects against *Plasmodium* and *Brugia* infections^5^,^8^,^29^ and contributes to nutrition^30^. Our findings add a detrimental effect by showing that bacterial multiplication following a blood meal exerts a strong negative impact on mosquito fitness affecting both mosquito survival and fecundity. Thus, although the microbiota is largely beneficial to its mosquito host, bacterial growth following a blood meal appears to mimic an oral infection in reducing mosquito fitness and, as shown before, inducing an immune response that limits their proliferation^5^,^8^. The combination of beneficial and detrimental effects of microbiota on its host reflects the coexistence of tolerance and resistance mechanisms towards gut bacteria^3^,^5^,^31^, which have both been found to antagonise invasion by malaria parasites^3^,^5^.

Sequencing of microbiota revealed that the near clearance of *Enterobacteriaceae* following PS ingestion correlates with an increase in *Acetobacteraceae*. This indicates a competition between bacteria taxa in the mosquito gut, where the niche cleared of some bacterial strains by antibiotic treatment is filled by others, as observed in human colon microbiota^32^. Specifically, we observed that *Serratia* was highly reduced after a PS-containing blood meal, whereas *Asaia* increased following treatment. These data may be of great relevance to the research towards engineering microbiota to block malaria transmission, as both bacteria are considered good paratransgenesis candidates^33^,^34^.

Of the antibiotics used in this study, penicillin inhibits peptidoglycan biosynthesis and targets mostly Gram-positive bacteria^35^, while streptomycin binds to the bacterial 30S ribosomal subunit, blocking protein synthesis in both Gram-positive and Gram-negative bacteria^36^. As our *Anopheles* colony microbiota principally consist of Gram-negative bacteria (only 0.17% of the reads belonged to Gram-positive bacteria), it is likely that the effects reported here are largely due to streptomycin. This antibiotic is used in tuberculosis treatments^11^ and is also part of the World Health Organization Buruli ulcer elimination program^12^. As Gram-negative bacteria are also dominant in field mosquitoes^20^, our data suggest that the extensive use of streptomycin in an area of high malaria endemicity may exaggerate disease transmission. The statistically non-significant increase of the infection prevalence of field *P. falciparum* populations upon PS treatment in our semi-field experiments, as opposed to the statistically significant increase in intensity, is likely due to the high infection intensities obtained and not to special attributes of the natural transmission system, where prevalence and intensity are shown to be closely linked. Similarly to the effect on fecundity, streptomycin treatment might impact mosquito infection prevalence early in the rainy season, when the gametocytemia is still low^37^,^38^.

Other antibiotics targeting the bacterial 30S ribosomal subunit, including doxycycline, are also commonly used in sub-Saharan Africa. Interestingly, doxycycline and azithromycin are used for malaria prophylaxis as they are known to impair *Plasmodium* asexual development; however, they do not reduce *Plasmodium* gametocyte numbers^39^,^40^ and have no clear direct effect on mosquito stages of *Plasmodium* development^17^. Therefore, whilst they reduce malaria morbidity, their overall impact on malaria transmission may require further investigation. Antibiotics such as trimethoprim and sulfamethoxazole directly impair *Plasmodium* development within the mosquito^17^ and may negatively or positively influence malaria transmission depending on the balance between their direct effects on *Plasmodium* and their indirect, microbiota mediated, effects on *Plasmodium* and mosquito fitness. Our data stresses the need for all these factors to be considered when studying antibiotics as malaria transmission-blocking candidates.

The implications of our findings for the global effort to reduce malaria transmission depend on the rate of antibiotic usage in malaria-endemic areas. Recent surveys in sub-Saharan African countries estimate antibiotic prescription to feverish children attending health centres at 43–71% and to feverish adults at 36% (refs 13, 14, 15). These frequencies are likely to increase in coming years due to intensified antibiotic treatment of diseases overlapping geographically and seasonally with malaria. Tuberculosis and leprosy patients, as well as HIV/AIDS patients living in high tuberculosis-burden areas, are prescribed long courses of antibiotics^10^,^11^, while mass administration of antibiotics for the elimination of neglected tropical diseases, such as mass azithromycin treatments against trachoma, are recommended by the World Health Organization or are currently under investigation^9^,^41^,^42^. Taken together, day-to-day antibiotic usage, long-lasting regimens and mass-targeted treatments result in high antibiotic incidence in human blood and may thus considerably impact malaria transmission.

Antibiotic usage including mass drug administration is shown to significantly reduce childhood mortality in malaria-endemic countries^43^. Our data suggest that understanding the impact of these antibiotics on malaria transmission may add significant value to such treatments. For example, prescription of an antibiotic shown to enhance malaria transmission could be combined with a recommendation for increased bednet usage, further reducing the exposure to mosquito bites. Such antibiotics could be alternatively co-prescribed with drugs such as atovaquone^42^, which not only combat malaria infection but also block its transmission. This is especially true for young children that are both highly vulnerable to malaria infection and very efficient at transmitting the parasite to mosquitoes^37^. If possible, antibiotics that do not promote or even prevent malaria transmission could be prioritised.

## Methods

### Ethics statement

All experiments performed in the United Kingdom were carried out in accordance with the United Kingdom Animals (Scientific Procedures) Act 1986. The protocol for infecting mosquitoes with *P. berghei* by blood feeding on parasite-infected mice was approved and carried out under the UK Home Office License PPL70/7185 awarded in January 2010. The procedures are of mild to moderate severity and the numbers of animals used are minimised by incorporation of the most economical protocols. Opportunities for reduction, refinement and replacement of animal experiments are constantly monitored and new protocols are implemented following approval by the Imperial College Ethical Review Committee.

All experiments performed in Burkina Faso were carried out in accordance with guidelines of the Centre Muraz Institutional Ethics Committee. The protocols for mosquito maintenance and for human blood collection were approved by the Centre Muraz Institutional Ethics Committee under the ethical clearance number 003-2009/cE-cM. Before enrollment, written informed consent was taken from each human volunteer and/or their legal guardian.

### Mosquito colony and maintenance

Most experiments were performed with female mosquitoes from an M form *A. gambiae* Ngousso colony, established from field mosquitoes collected in Cameroon in 2006, maintained on human blood and fed as adults with 5% fructose. The field parasite infections were performed with female mosquitoes from an S form *A. gambiae* colony established from field mosquitoes collected in Burkina Faso in 2009^24^, maintained on rabbit blood and fed as adults with 5% glucose. Larvae were fed tetramin fish food. Insectary temperature was maintained at 28 °C (±1 °C), 70–80% humidity on a 12 h light/dark cycle.

### Human blood feeding and *Plasmodium* infections

*P. falciparum* NF54 gametocytes were cultured in Roswell Park Memorial Institute medium supplemented with 50 mg l^−1^ hypoxanthine, 10% heat-inactivated human serum in the absence of antibiotics. Two 25 ml cultures were started 14 and 17 days before the infection with 6% v/v washed O^+^ red blood cells (RBCs) infected at 0.5% and media was changed every day. Before mosquito infection, centrifuged RBCs were pooled and supplemented with fresh RBCs and human serum (final concentrations: 32% cultured RBCs, 8% fresh RBCs, 60% serum). For experiments carried out from field parasites, gametocyte carriers were selected among 5–14-year-old school children in Burkina Faso. Vein blood was sampled into a lithium-heparin coated tube and serum was replaced by European AB serum to reduce the chance of transmission-blocking immunity. Mosquitoes were offered a blood meal from a membrane-feeding device covered with parafilm and kept at 37 °C. Twenty microlitres of a cocktail containing 1,000 μg ml^−1^ streptomycin plus 1,000 U ml^−1^ penicillin (Sigma) was added to 1 ml of blood. The final streptomycin concentration in the blood (20 μg ml^−1^) is chosen as a proxy to the average therapeutic concentration upon treatment^44^,^45^, while the concentration of penicillin (20 U ml^−1^) is within the recommended range of therapeutic concentrations that vary substantially^46^. Control blood was supplemented with 20 μl of water. For *P. berghei* infections, 8–12-week-old TO female mice were intraperitoneally injected with 100 μl EF1 *P. berghei*-infected blood. Two days later, a group of four to five mice with 3–8% parasitemia was anaesthetised by 4 μl g^−1^ intramuscular injection of Rompun (0.33%) and ketamine (33 mg ml^−1^) in PBS and used to blood feed a group of mosquitoes for 10–15 min. To reduce the mice inter-individual effects, the same mice were intravenously injected with 2 μg g^−1^ streptomycin, 2 U g^−1^ penicillin, left for 10 min for antibiotic diffusion in the bloodstream (20 μg ml^−1^ streptomycin, 20 U ml^−1^ penicillin after diffusion in a volume of 92 ml kg^−1^ of blood^47^) and used to feed a second group of mosquitoes for 10–15 min. For each *Plasmodium* infection, at least 100 mosquitoes per condition were offered a blood meal and non-engorged mosquitoes were removed. All mosquitoes that blood fed and survived until the end of the experiment were dissected for oocyst counts using mercurochrome staining (*P. falciparum*) or fluorescence (*P. berghei*). On average, the oocysts of 86 (±8 s.e.m.) mosquitoes per condition per experiment were counted.

### Microbiota analysis by qPCR

Non-blood-fed mosquitoes were discarded after the blood meal. Before dissection, mosquitoes were immersed in 75% ethanol for 5 min to kill mosquitoes and to fix surface bacteria to the cuticle. Mosquitoes were then washed three times in sterile PBS to wash out non-attached bacteria, thus preventing sample contamination with cuticle bacteria during dissection. Midguts were removed and frozen immediately on dry ice in three groups of at least five mosquitoes per condition and stored at −20 °C until processing. Midguts were only excluded from analysis if they burst and that a substantial amount of the gut content was lost. Samples were homogenised with 0.5-mm wide glass beads (Bertin) for 30 s at 6,800 r.p.m. in a Precellys 24 homogenizer (Bertin) in phenol–chloroform and DNA was extracted with phenol–chloroform. The 16S rDNA was used for bacterial quantification and is shown as a ratio of the *Anopheles* housekeeping gene *40S ribosomal protein S7* (VectorBase gene ID AGAP010592), referred to as S7. Primer sequences are listed in Supplementary Table 3. qPCR was performed on a 7,500 Fast Real-Time thermocycler (Applied Biosystems) using the SYBR *Premix Ex Taq* kit (Takara), following the manufacturer’s instructions.

### Microbiota analysis by 454-pyrosequencing

Mosquitoes were treated as for qPCR analysis, with special care to reduce risks of contamination: dissecting slides and forceps were washed with detergent, rinsed with water and then with ethanol between each sample and sample-containing tubes were ultraviolet pretreated. At least 15 mosquitoes per condition were dissected. A contamination control was included, where drops of PBS were left on a dissecting slide and then transferred 10 times with forceps into the tube to mimic dissection routine. DNA extraction conditions were chosen according to Yuan *et al*.^48^ to sample the microbiota composition as accurately as possible. Samples were homogenised for 15 s in a lysis buffer (20 mM Tris pH 8.0, 2 mM EDTA, 1.2% Triton 20 mg ml^−1^ lysozyme, 250 U ml^−1^ mutanolysin and 20 U ml^−1^ lysostaphin), incubated for 30 min at 37 °C and homogenised again for 15 s. DNA was extracted using a DNeasy blood and tissue kit (Qiagen) under sterile conditions. qPCR confirmed that this extraction method provided a similar pattern of bacterial growth that is reduced in presence of PS as seen with phenol–chloroform (Fig. 1a, Supplementary Fig. 3). The regions V2-V3 of the 16S rDNA were amplified using 16S–0027F (5′-AGAGTTTGATCCTGGCTCAG-3′ ) and 16S–0533R (5′-TTACCGCGGCTGCTGGCAC-3′ ) attached in 5′ to Roche MID adapters (Supplementary Table 4) with Phusion polymerase (New England Biolabs; 27 cycles: 5 s at 98 °C, 20 s at 63 °C, 20 s at 72 °C, followed by 5 min at 72 °C). After Picogreen (Life Technologies) quantification, a pool of 48 samples (including the 24 samples reported here), each of 50 ng DNA, was 454-pyrosequenced^49^ using a half run of a 454 GS FLX sequencer (Roche) by Beckman Coulter Genomics. Sequencing resulted in 544038 sequences processed with Qiime^50^ (version 1.8.0). Of these, 177287 reads of 477 bp average length after primer truncation were assigned to the 24 samples (no primer mismatch allowed), and 175627 of them were identified as non-chimeric using usearch61^51^ (see Supplementary Table 4 for details on each sample). operational taxonomic units (OTUs) were picked using uclust software with a 97% identity threshold^51^. Among the contamination controls, one replicate produced only 12 reads after filtering; the other two were significantly different from mosquito gut samples (ANOSIM test output *P*=0.024 and *P*=0.004 on weighted and unweighted Unifrac distances^52^, respectively) and were thus excluded from subsequent analyses. Alpha diversity was quantified using the number of observed species, the Chao1 species richness estimation^53^, the Shannon index and the phylogenetic diversity whole-tree index^54^ (Supplementary Table 1). OTUs were assigned to taxonomy against the Silva reference data set (Release 119)^55^. Principal coordinates analysis was performed using Emperor^56^.

### Blood meal size quantification

Individual females were housed in plastic vials after blood feeding and allowed to deposit their faecal pellets. All excreted material was dissolved in 1 ml 1% lithium carbonate solution and the absorbance measured at 387 nm^57^. The quantity of haematin in each faecal pellet was calculated by comparison with a standard curve constructed with porcine serum haematin (Sigma). At least eight mosquitoes per condition were used for each replicate.

### Survival

Age-matched 2–6-day-old mosquitoes were offered a control or experimental blood meal once (Fig. 3c) or every 4 days (Fig. 3b) for a total of three blood meals. Between the blood meals, mosquitoes were provided with a cotton pad moistened with distilled water for oviposition. Unfed mosquitoes were removed after each blood meal and excluded from data analysis. This did not impact survival data, as blood treatment did not significantly affect the number of unfed mosquitoes after the second and third blood meals (*P*=0.60, ANOVA/glmer). Deaths were counted and carcasses removed on a daily basis. At least 50 mosquitoes per condition were used for each replicate.

### Fecundity and fertility

Blood-fed mosquitoes were stored in individual cups with filter paper moistened with distilled water for oviposition. For fecundity analyses, eggs were counted upon laying. Females that had not laid eggs after at least 6 days were considered non-egg layers. For fertility analyses, eggs were counted and floated in salt water. Larvae were counted and removed on a daily basis. Any egg remaining after 1 week was considered unhatched. At least 50 mosquitoes per condition were used for each replicate.

### Statistical analyses

Statistical analyses were performed by generalized linear mixed models (GLMM) in R (version 2.15.3). Factorial data (prevalence, egg laying and proportion of unfed mosquitoes for survival experiments) were analysed by ANOVA *χ*^2^-test on a logistic regression (glmer). Oocyst counts were analysed by Wald *Z*-test on a zero-inflated negative binomial regression (glmmADMB). 16S data (qPCR, alpha diversity, family relative abundance from pyrosequencing) and egg counts were analysed by ANOVA *χ*^2^-test on a common linear regression (lmer). GLMM analyses decompose model effects into the contribution of a fixed component (the covariates in question, here treatments) and a random component (here experiments for each treatment). The fixed effect estimates are the regression coefficients. For infection intensity we report both the fixed effects and the variations in the median number of oocysts per mosquito. Human and mouse blood meal survival data were analysed using a log-rank test and a Gehan–Wilcoxon test, respectively, to reflect the experimental setup where human blood meals are given throughout the experiment and a mouse blood meal only on the first day. In both the cases, survival tests were stratified by experimental replicate. All differences between PS-treated and control mosquitoes are calculated as means of all proportional changes in the independent replicates; that is, (data(PS)−data(control))/data(control) expressed as a percentage. Sample size was chosen according to the standard protocols (oocyst counts, blood meal quantification, survival, fecundity and fertility) or following preliminary tests on individual mosquitoes (microbiota analyses). Mosquitoes were excluded if they did not blood feed, if they burst during dissection (microbiota analyses) or if they died during the experiment (except for survival experiments).

## Additional information

**How to cite this article**: Gendrin, M. *et al*. Antibiotics in ingested human blood affect the mosquito microbiota and capacity to transmit malaria. *Nat. Commun.* 6:5921 doi: 10.1038/ncomms6921 (2015).

**Accession codes:** The 16S sequencing data have been deposited in the European Nucleotide Archive under accession code PRJEB7708.

## Supplementary Material

Supplementary Information Supplementary Figures 1-3, Supplementary Tables 1-4, and Supplementary References
