# Supplementary material for: Antibiotics in ingested human blood affect the mosquito microbiota and capacity to transmit malaria
Source: Nat Commun. 2015 Jan 6;6:5921. doi: 10.1038/ncomms6921 (PMC4338536; doi:10.1038/ncomms6921)
Supplement: Supplementary Information — Supplementary Figures 1-3, Supplementary Tables 1-4, and Supplementary References [file ncomms6921-s1.pdf]

Supplementary Information

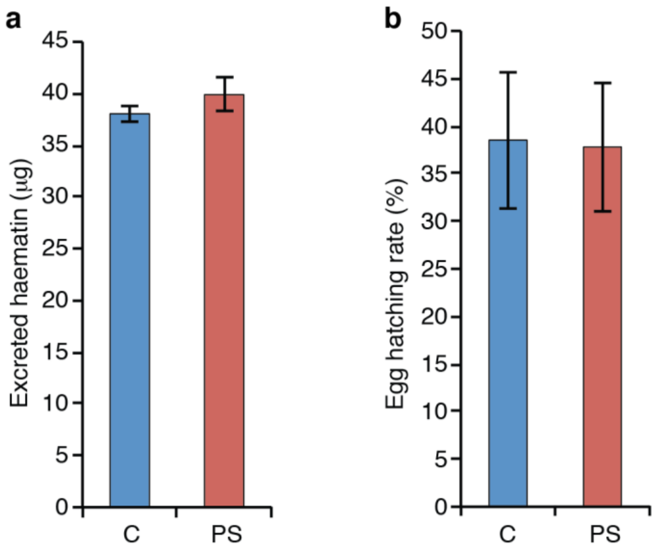

Supplementary Fig. 1. Bloodmeal antibiotics do not affect the bloodmeal size and egg hatching rate.

The bloodmeal size (**a**) and egg hatching rate (**b**) were quantified in mosquitoes fed on blood supplemented with PS or water (control, 'C'). In **a**, the bloodmeal size was quantified by haematin excretion revealed by colorimetric reaction with $LiCO_3$. Results are shown as averages of 2 (**a**) and 3 (**b**) independent experiments and error bars show data range (**a**) and standard errors (**b**). n=8-12 (**a**) and 50-80 (**b**) mosquitoes per sample

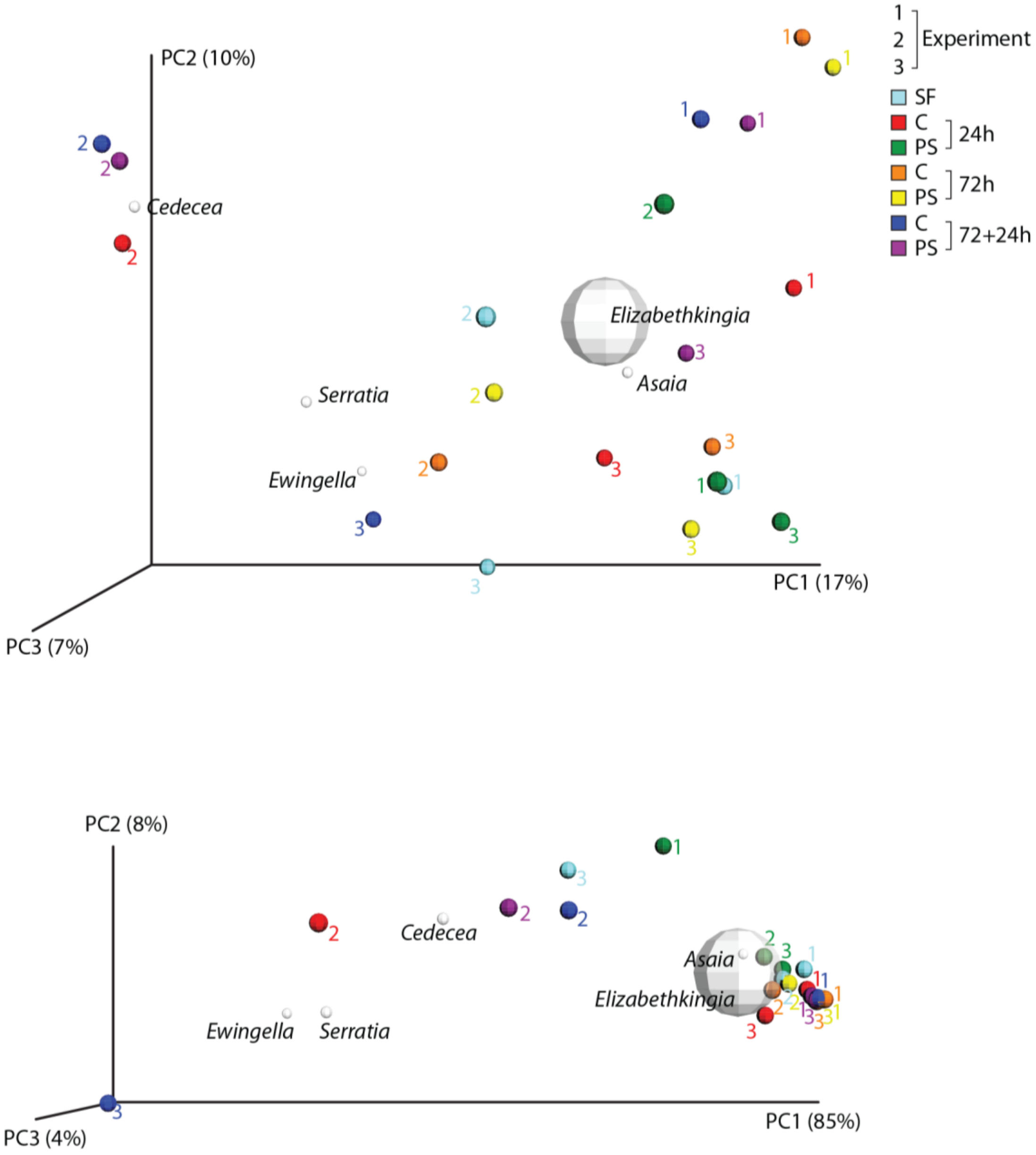

Supplementary Fig. 2. Principal Coordinates Analysis of the microbiota composition. The PcoA is based on unweighted (top) and weighted (bottom) Unifrac distances between the samples included in the pyrosequencing analysis (**Fig. 1b**). Each coloured sphere represents one sample, its colour and number reflecting its feeding condition and replicate, respectively. Grey spheres are localised where the five main bacterial genera are more prevalent and their size reflects their contribution to the microbiota composition. n=15-25 mosquitoes per sample

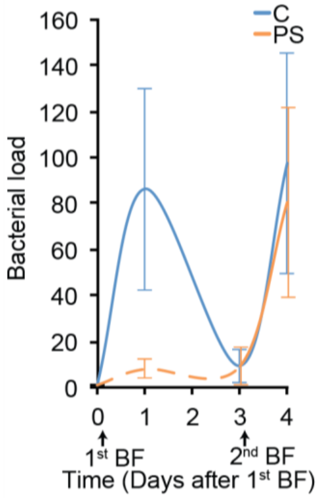

Supplementary Fig. 3. qPCR analysis of the samples included in the pyrosequencing experiment. qPCR quantification of midgut bacterial 16S rDNA in sugar-fed mosquitoes, 24h and 72 h after blood-feed supplemented with PS or water and 24h after a second non-treated blood-feed. Data show the average [± SEM] of three independent experiments included in the pyrosequencing analysis shown **Fig. 1b**. n=15-25 mosquitoes per sample.

Supplementary Table 1. Alpha diversity observed by 454-pyrosequencing

	24h			72h			72h+24h		
	C	PS	p-value	C	PS	p-value	C	PS	p-value
Chao1	178.5	92.6	0.00030	118.2	96.9	0.041	191.4	161.0	0.10
Shannon	1.27	0.84	0.022	0.28	0.25	0.0015	1.96	1.01	0.00039
Observed Species	90.2	33.4	2.9E-6	32.3	28.8	0.0013	104.5	71.9	0.0017
PD whole tree	8.76	4.20	2.3E-6	4.30	4.05	0.053	9.67	6.91	0.00093

C - Control; PS - PS-treated; PD: phylogenetic diversity. Statistical tests: ANOVA/lmer.

Supplementary Table 2. Detailed results of infections with naturally *P. falciparum*-infected blood

	Experiment 1		Experiment 2		Experiment 3		Experiment 4	
	C	PS	C	PS	C	PS	C	PS
**Gametocytemia**	16	16	20	20	208	208	160	160
**Number of mosquitoes**	36	40	18	31	81	100	54	48
**Prevalence**	63.9	77.5	77.8	90.3	86.4	90.5	70.4	58.3
**Median (oocysts)**	1.0	3.0	2.5	8.0	20.0	32.0	4.0	2.0
**Average (oocysts)**	3.1	4.5	3.5	7.8	29.3	39.2	6.3	5.2
**Average excluding 0 (oocysts)**	4.9	5.8	4.5	8.6	33.9	43.3	8.9	8.9

Gametocytemia shows the number of gametocytes per 1000 leucocytes.

Supplementary Table 3. Primer sequences used for qPCR analysis

Target	Forward primer	Reverse primer
Eubacteria [1]	TCCTACGGGAGGCAGCAGT	GGACTACCAGGGTATCTAATCCTGTT
Flavobacteriaceae	TAAGGTTGAAGTGGCTGGAATAA	GTCCATCAGCGTCAGTTAAGACT
Enterobacteriaceae	CGTGCTACAATGGCATATACAAAGAGAAG	AGCATTCTGATCTACGATTACTAGCGATTC
Acetobacteraceae	GTGCCGATCTCTAAAAGCCGTCTCA	TTCGCTCACCGGCTTCGGGT
***A. gambiae* S7**	GTGCGCGAGTTGGAGAAGA	ATCGGTTTGGGCAGAATGC

Supplementary Table 4. 454-pyrosequencing multiplex identifiers (MID) and number of sequences obtained for each sample

Expe-riment	Treatment	MID Number	Barcode Sequence	All sequences	Non-chimeric sequences
1	SF	6	ATATCGCGAG	10739	10369
1	BF C 24h	8	CTCGCGTGTC	9231	9229
1	BF PS 24h	9	TAGTATCAGC	7744	7486
1	BF C 72h	12	TACTGAGCTA	10798	10787
1	BF PS 72h	13	CATAGTAGTG	13816	13815
1	BF C 72+24h	17	CGTCTAGTAC	14195	14185
1	BF PS 72+24h	18	TCTACGTAGC	8723	8683
1	negative control	21	CGTAGACTAG	3476	3222
2	SF	22	TACGAGTATG	6549	6389
2	BF C 24h	24	TAGAGACGAG	8138	8136
2	BF PS 24h	25	TCGTCGCTCG	4559	4358
2	BF C 72h	28	ACTACTATGT	2773	2757
2	BF PS 72h	29	ACTGTACAGT	6757	6723
2	BF C 72+24h	33	ATAGAGTACT	4873	4873
2	BF PS 72+24h	34	CACGCTACGT	8960	8956
2	negative control	37	TACACACACT	14	14
3	SF	38	TACACGTGAT	8240	8213
3	BF C 24h	40	TACGCTGTCT	10354	10282
3	BF PS 24h	41	TAGTGTAGAT	9361	9355
3	BF C 72h	44	TCTAGCGACT	8060	8060
3	BF PS 72h	45	TCTATACTAT	9411	9411
3	BF C 72+24h	49	ACGCGATCGA	3731	3704
3	BF PS 72+24h	50	ACTAGCAGTA	4866	4863
3	negative control	53	AGTCGAGAGA	1919	1757

Supplementary reference

1 Nadkarni, M. A., Martin, F. E., Jacques, N. A. & Hunter, N. Determination of bacterial load by real-time PCR using a broad-range (universal) probe and primers set. *Microbiology* **148**, 257-266 (2002).
